# Microsecond Molecular Dynamics Simulations of Intrinsically Disordered Proteins Involved in the Oxidative Stress Response

**DOI:** 10.1371/journal.pone.0027371

**Published:** 2011-11-18

**Authors:** Elio A. Cino, Jirasak Wong-ekkabut, Mikko Karttunen, Wing-Yiu Choy

**Affiliations:** 1 Department of Biochemistry, The University of Western Ontario, London, Ontario, Canada; 2 Department of Applied Mathematics, The University of Western Ontario, London, Ontario, Canada; 3 Faculty of Science, Kasetsart University, Chatuchak, Bangkok, Thailand; 4 Department of Chemistry, University of Waterloo, Waterloo, Ontario, Canada; Governmental Technical Research Centre of Finland, Finland

## Abstract

Intrinsically disordered proteins (IDPs) are abundant in cells and have central roles in protein-protein interaction networks. Interactions between the IDP Prothymosin alpha (ProTα) and the Neh2 domain of Nuclear factor erythroid 2-related factor 2 (Nrf2), with a common binding partner, Kelch-like ECH-associated protein 1(Keap1), are essential for regulating cellular response to oxidative stress. Misregulation of this pathway can lead to neurodegenerative diseases, premature aging and cancer. In order to understand the mechanisms these two disordered proteins employ to bind to Keap1, we performed extensive 0.5–1.0 microsecond atomistic molecular dynamics (MD) simulations and isothermal titration calorimetry experiments to investigate the structure/dynamics of free-state ProTα and Neh2 and their thermodynamics of bindings. The results show that in their free states, both ProTα and Neh2 have propensities to form bound-state-like β-turn structures but to different extents. We also found that, for both proteins, residues outside the Keap1-binding motifs may play important roles in stabilizing the bound-state-like structures. Based on our findings, we propose that the binding of disordered ProTα and Neh2 to Keap1 occurs synergistically via preformed structural elements (PSEs) and coupled folding and binding, with a heavy bias towards PSEs, particularly for Neh2. Our results provide insights into the molecular mechanisms Neh2 and ProTα bind to Keap1, information that is useful for developing therapeutics to enhance the oxidative stress response.

## Introduction

IDPs are a class of proteins that are biologically functional despite lacking well-defined structures [1]–[5]. They are abundant in nature: 25–30% of eukaryotic proteins are predicted to be at least partially disordered, while up to 70% of signaling proteins may contain intrinsically disordered regions [6], [7]. Compared to globular proteins, the amino acid compositions of IDPs are usually biased towards charged, polar and structure-breaking residues, such as glycine and proline [3], [8], [9]. As a result, in the absence of binding partners, these proteins generally lack structured hydrophobic cores and display high conformational flexibility [3], [5].

Despite their dynamic nature, IDPs seldom adopt completely random coil conformations [10]–[13]. In fact, many IDPs are found to possess considerable conformational propensities along their sequences [14]–[20]. These transiently structured regions frequently act as molecular recognition features for target binding [16], [18], [21], [22]. Interestingly, interactions with different partners can also cause a disordered region to adopt distinct conformations [2], [18], [21], [23]. For example, the same region of the intrinsically disordered C-terminus of p53 can adopt either a helix or a β-strand structure depending on the target it interacts with [23]. These unique structural properties empower many IDPs to act as hubs in protein-protein interaction networks through low-affinity but yet highly specific binding [4], [21], [24]–[26]. Therefore, it is not a surprise that IDPs are frequently associated with human diseases, in particular cancer and neurodegenerative diseases [27]–[29].

Even though IDPs are involved in crucial biological functions, the mechanisms by which they interact with targets are not well understood. Recent studies have shown that some IDPs undergo large conformational changes upon target binding [4], [30]–[32], while others have preformed structural elements (PSEs) that resemble the bound state conformations in a significant population of conformers in the ensemble [16], [33]–[35]. It is noteworthy that these two mechanisms are not always independent; in many cases, the binding of IDPs to their targets involves a combination of both [36]. Knowledge of the detailed mechanisms that IDPs employ to bind to their targets is critical for understanding how this class of proteins function. More importantly, it will also aid in the development of therapeutic agents targeting these types of interactions [37], [38].

While X-ray crystallography is commonly used to determine protein structures with atomic-level accuracy, the dynamic nature of IDPs makes acquiring diffracting crystals of these proteins in free states extremely challenging [2]. Nuclear magnetic resonance (NMR) spectroscopy has become the primary technique for the structural characterization of this class of proteins [39], [40]. Despite the fact that NMR can yield a wealth of data, there are limitations. For an IDP undergoing fast conformational exchange on the NMR timescale, collected data are averaged over the entire ensemble of conformations sampled by the protein. Therefore, unlike for folded proteins, it is inappropriate to determine a single conformation to represent the disordered state. To circumvent this problem, molecular dynamics (MD) simulations have been used to complement the experimental techniques in order to establish better models for describing the dynamic nature of interconverting disordered state ensembles and, more importantly, the mechanisms by which IDPs interact with targets. For instance, MD simulations have been performed on both the bound and apo phosphorylated forms of intrinsically disordered kinase-inducible domain (KID) to investigate the molecular mechanism by which pKID interacts with KIX in signal transduction [41]. Wu *et al.* have combined NMR spectroscopy and MD simulations to identify the structural reorganization of alpha-synuclein at low pH [42].

The objective of this work is to understand the molecular mechanisms that the disordered ProTα and Neh2 domain of Nrf2 use to bind Keap1 in the oxidative stress response pathway. Exposure to toxic reactive electrophiles from the environment as well as those generated by our own metabolism can disrupt the cellular functions, resulting in neurodegenerative diseases, cancer and aging [43]. Nrf2 is a key transcription factor for genes responsive to oxidative stress [44], [45]. The protein consists of six highly homologous regions (Neh1-6 domains). The Neh2 domain, which is located at the N-terminus of Nrf2, plays a regulatory role by interacting with an ubiquitously expressed inhibitor, Keap1 [45]. Under homeostatic conditions, the Neh2 domain of Nrf2 binds to the Kelch domains of the monomeric units of a Keap1 dimer via a high affinity ETGE motif and a lower affinity DLG motif (with K_d_ values of ∼8 nM and ∼0.5 µM), respectively [46]. When both motifs are bound to a Keap1 dimer, Neh2 is (poly) ubiquitinated and subsequently degraded by the proteosome [45]–[48]. When the cells are under oxidative stress conditions, the interaction of Keap1 and Nrf2 is disrupted, leading to the upregulation of Nrf2-mediated gene expression.

**Figure 1:**
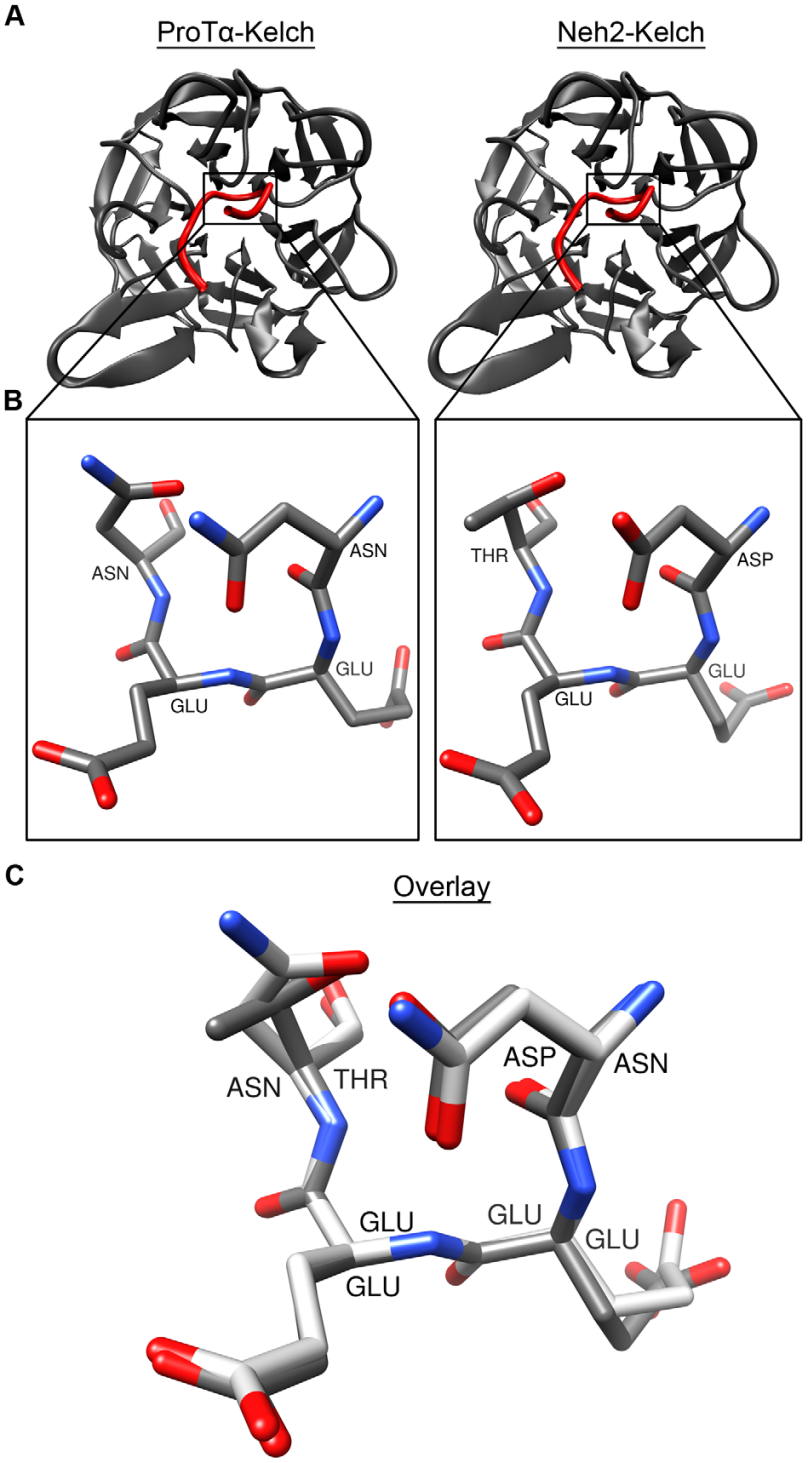
Crystal structures of ProTα and Neh2 peptides bound to the Kelch domain of Keap1. A) Cartoon B-Spline representations of the ProTα-Keap1 and Neh2-Keap1 crystal structures (PDB ids: 2Z32 and 1X2R respectively [50], [55]. Residues Asn-41 to Glu-48 of ProTα and Leu-76 to Leu-84 of Neh2 (red) are shown bound to the Kelch domain of Keap1 (grey). B) Licorice representations of the *i* to *i*+3 residues of the β-turns from the crystal structures (^41^Asn-Glu-Glu-Asn^44^ and ^77^Asp-Glu-Glu-Thr^80^, of ProTα and Neh2 respectively). C) Overlay of the ProTα (white) and Neh2 (grey) β-turns.

Recent studies have shown that ProTα can compete with Nrf2 for binding to Keap1, resulting in the upregulation of Nrf2-targeted cytoprotective genes [49], [50]. ProTα is ubiquitously expressed in a wide variety of human tissues and besides the regulatory role it plays in the expression of oxidative stress response genes, the protein has also been found to be involved in other cellular processes such as cell proliferation, chromatin remodeling, transcriptional regulation and apoptosis [51]–[53]. The Keap1-binding motif of ProTα (-NEENGE-) shares a similar sequence with that of the Neh2 (-DEETGE-). Crystal structures of ProTα and Neh2 peptides bound to the Kelch domain of Keap1 further reveal that these two proteins bind to the same site on the Kelch domain and form similar β-turn conformations [46], [50] (Figure 1). The Kelch domain adopts a six-bladed β-propeller structure with each blade composed of four anti-parallel β-strands [46], [50]. Both ProTα and Neh2 bind to the positively charged face of the β-propeller where the inter-blade loops are located and the electrostatic interactions are crucial for the stability of the complexes [46], [50]. Interestingly, despite the high sequence identity and structural similarity of the binding motifs, ProTα seems to have a lower binding affinity to Keap1 (see result below) compared to Neh2 (only the ETGE motif is considered) [46], [49].

Atomistic microsecond scale MD simulations were used to investigate the molecular mechanisms by which the intrinsically disordered ProTα and Neh2 interact with Keap1. In particular, we focused on whether their XEEXGE motifs bind to Kelch domain through coupled folding and binding, PSEs or a combination of both mechanisms. Our results show that in their free states, both the Keap1-binding motifs of ProTα and Neh2 display intrinsic propensities to form bound-state-like β-turns, and that the residues outside of the motifs may also contribute to the stability of the structural elements. We found that the Keap1-binding motif of Neh2 adopted a β-turn conformation that more closely resembled its bound-state structure than that of ProTα. Based on these results, we propose that binding occurs synergistically via a combination of PSEs and coupled folding and binding with a heavy bias towards PSEs, especially for Neh2. The better understanding of the binding mechanisms may provide insight into developing of therapeutics that can be used to promote cellular response to oxidative stress.

## Materials and Methods

### Starting structures

The free state structure and dynamics of ProTα and Neh2 were investigated using atomistic MD simulations. All starting structures were generated using the Crystallography & NMR System (CNS) software package [54]. Briefly, extended structures were first generated based on the amino acid sequences of ProTα and Neh2. Each structure subsequently underwent a simulated annealing simulation using default CNS parameters from the anneal.inp script [54]. By using this procedure, we generated structures of peptides with identical sequences and lengths to those used to generate the crystal structures of mouse ProTα and Neh2 bound to Keap1 (PDB ids: 2Z32 and 1X2R respectively) [50], [55], the full-length mouse ProTα protein and a 32-mer mouse Neh2 peptide, as well as their human homologs. Table 1 summarizes the amino acid sequences used in the MD simulations and the lengths of the trajectories. Peptides with longer sequences (full-length ProTα protein and the 32-mer Neh2 peptide) were simulated to determine if residues outside of the Keap1 binding motif might be important for binding, while human sequences were simulated for cross-species comparison. To focus on the ETGE binding motif, the 32-mer Neh2 peptides instead of the full-length proteins were simulated in order to exclude the N-terminus DLG motif and the central helical region, which is not involved in Keap1 binding [46]. To avoid biasing the sampling towards native-like conformations, conformers from the annealing simulations that did not resemble their bound-states were chosen as starting structures (Figure S1). The underlined residues in Table 1 comprise the Keap1-binding β-turns of ProTα and Neh2, determined from the crystal structures [50], [55], and are referred to as positions *i* through *i*+3 in this work (Figure 1).

**Table 1:** Amino acid sequences of the simulated molecules and trajectory lengths.

System	Sequence	Simulation time (µs)
16-mer ProTα peptide (mouse)	^39^AQNEENGEQEADNEVD^54^	1.0
9-mer Neh2 peptide (mouse)	^76^LDEETGEFL^84^	1.0
Full-length ProTα (mouse)	^1^MSDAAVDTSSEITTKDLKEKKEVVEEAENGRDAPANGNAQNEENGEQEADNEVDEEEEEGGEEEEEEEEGDGEEEDGDEDEEAEAPTGKRVAEDDEDDDVDTKKQKTEEDD^111^	0.5
32-mer Neh2 peptide (mouse)	^69^AFFAQFQLDEETGEFLPIQPAQHIQTDTSGSA^100^	0.5
Full-length ProTα (human isoform 2)	^1^MSDAAVDTSSEITTKDLKEKKEVVEEAENGRDAPANGNANEENGEQEADNEVDEEEEEGGEEEEEEEEGDGEEEDGDEDEEAESATGKRAAEDDEDDDVDTKKQKTDEDD^110^	0.5
32-mer Neh2 peptide (human isoform 1)	^69^AFFAQLQLDEETGEFLPIQPAQHIQSETSGSA^100^	0.5

Residues *i* through *i*+3 of the β-turn regions of the ProTα and Neh2 sequences, determined from the crystal structures [50], [55] are underlined.

### MD simulations

All simulations were performed using GROMACS (GROningen MAchine for Chemical Simulations) version 4 [56], with the GROMOS96 53a6 united atom force-field parameter set [57], [58]. This force field has been shown to be reliable in simulating proteins, including β-peptide folding [59]. Protonation states of ionizable residues were chosen based on their most probable state at pH 7. The amino and carboxyl terminals of all systems were capped with NH_3_^+^ and COO^−^ groups respectively. The starting structures were solvated in simple point charge (SPC) water [60], followed by the addition of sodium (Na^+^) and chloride (_Cl_^−^) ions to make the system charge neutral and bring the salt concentration to 0.1 M. The 16-mer ProTα and the 9-mer Neh2 systems (Table 1) contained between 9950 and 5926 water molecules and 43 to 26 molecules of salt, respectively. The full-length ProTα and the 32-mer Neh2 systems (Table 1) contained between 68146 and 16887 water molecules and 293 to 67 molecules of salt, respectively. The GROMOS parameterization of Na^+^ and _Cl_^−^ was used, which has been shown to work well with SPC water [61]. MD simulations were performed at constant temperature, pressure and number of particles (NPT ensemble). Protein and non-protein atoms were coupled to their own temperature baths, which were kept constant at 310 K using the weak coupling algorithm [62]. Pressure was maintained isotropically at 1 bar using the Berendsen barostat [62]. Prior to the production runs, the energy of each system was minimized using the steepest descents method. This was followed by 2 ps of position-restrained dynamics with all non-hydrogen atoms restrained with a 1000 kJ mol^−1^ force constant. The timestep was set to 2 fs. Initial atom velocities were taken from a Maxwellian distribution at 310 K. All bond lengths were constrained using the LINCS algorithm [63]. Cut-off of 1.0 nm was used for Lennard-Jones interactions and the real part of the long-range electrostatic interactions, which were calculated using the Particle-Mesh Ewald (PME) method [64]. 0.12 nm grid-spacing was used for PME. It is important to treat electrostatic interactions with accurate methods, such as PME, to avoid potential serious artifacts [65], [66]. It has been shown that choosing simulation parameters, including thermostat and electrostatic treatment, is a subtle issue and that the choice of charge-groups may lead to unphysical effects [67]. Baumketner *et al*. [68], [69] also reported that charge-group based truncation with reaction-field electrostatics may cause artificial repulsions between charged residues, identified as the microscopic reason behind artificial unfolding of protein in some simulations. Here, charge-groups were chosen to be small to avoid artifacts [67]. Periodic boundary conditions were applied in all directions. This simulation protocol has been successfully applied in a number of our previous protein and membrane simulations [67], [70], [71]. Simulations of the shorter peptide systems took ∼1–2 weeks each using 32 cores, while the larger systems each took ∼3–7 weeks using 64 cores. The cumulative simulation time for all of the trajectories was ∼231, 000 CPU hours.

### Simulation analysis

To determine whether the binding motifs of ProTα and Neh2 have tendencies to adopt bound-state-like structures in their free states, coordinates from the MD trajectories were compared with the corresponding PDB crystal structures (PDB ids: 2Z32 and 1X2R respectively) [50], [55]. Distance-based root-mean-square deviations (RMSD) were computed between structures at time *t* of the trajectory and the bound state reference determined from the crystal structure using the equation [56]: 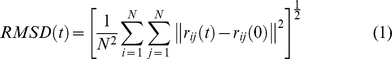



where *r_ij_*(t) and *r_ij_*(0) are the distances between atoms *i* and *j* at time *t* of the trajectory and the same pair of atoms in the bound-state structure, respectively.

The *C* α*^i^−C* α*^i+3^* distances were calculated to determine if Keap1-binding β-turns of ProTα and Neh2 were formed during the simulations. To be defined as a β-turn, the *C* α*^i^−C* α*^i+3^* distance must be less than 7 Å [72]. Residue specific dynamics of the β-turns were also probed by analyzing the circular variance (*C.V.*) of the ϕ and ψ dihedral angles over time. The *C.V.* is defined as [73]: 
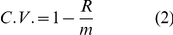



where *m* is the number of structures included in the analysis, and *R* is calculated using the following equation [73]: 
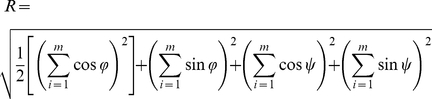
(3)


The value of *C.V.* ranges between 0 and 1. Lower values represent tighter clustering about the mean and higher values are indicative of greater ϕ and ψ variability.

Hydrogen bonds were analyzed as follows: A hydrogen bond between a donor (D–H) and an acceptor (A) was considered to be formed when the DA distance was less than 3.2 Å and the angle between the DA vector and the D–H bond (AD-H angle) was less than 35°[74], [75]. Visualization of the structures was done using VMD [76] and Chimera [77].

### Isothermal titration calorimetry (ITC) experiments

The Kelch domain (residues 324–612) of mouse Keap1 was expressed in *Escherichia coli* BL21 (DE3) grown in minimal M9 medium. The N-terminally His-tagged protein was purified by affinity chromatography using Ni Sepharose™ 6 Fast Flow beads (Amersham Biosciences). The tag was then cleaved by incubation with His-tagged tobacco etch virus (TEV) protease overnight at 25°C. The protein product was purified by passing the mixture through Ni Sepharose™ 6 Fast Flow beads (Amersham Biosciences).

ITC experiments were performed on a VP-ITC system (MicroCal) at 25°C. The Kelch domain was dialyzed against 50 mM phosphate buffer at pH 7, containing 100 mM NaCl and 1 mM DTT. Mouse ProTα (Ala-39 to Asp-54) and Neh2 (Leu-76 to Leu-84) peptides (GenScript) were also dissolved in the same buffer. All samples were filtered and degassed before the ITC experiments. Typically, 5 µL aliquots of 0.5 mM ProTα or Neh2 peptide were titrated to the sample cell containing 1.4 mL of 0.05 mM Kelch at 4-minute intervals. Heat changes after saturation were used to account for the heat of dilution. The binding stoichiometries (n), enthalpy changes (ΔH), binding constants (K_a_), Gibbs free energy changes (ΔG) and entropy changes (ΔS) were calculated using the titration data.

## Results and Discussion

MD simulations were used to study the free-state structure and dynamics of ProTα and the Neh2 domain of Nrf2. The crystal structures revealed that the NEENGE and DEETGE motifs of ProTα and Neh2, respectively, bind to same site on the C-terminal Kelch domain of Keap1 [50], [55] (Figure 1). In particular, both the segments NEEN and DEET of ProTα and Neh2 occupied positions *i* through *i+3* of their respective β-turns and adopted highly similar structures in their bound states (Figure 1). We compared the structures of free-state ProTα and Neh2 peptides from the MD simulations to their corresponding bound-state conformations [50], [55] in order to determine whether ProTα and Neh2 interact with Keap1 via PSEs or coupled folding and binding mechanisms. MD simulations on the full-length ProTα protein and a 32-mer Neh2 peptide were also performed to determine if the residues outside the binding motifs play a role in binding. Finally, contributing factors to the β-turn propensities of ProTα and Neh2 were investigated through circular variance, C_α_−C_α_ contact, and hydrogen-bond analyses.

### Comparison of the free and bound-state structures

**Figure 2:**
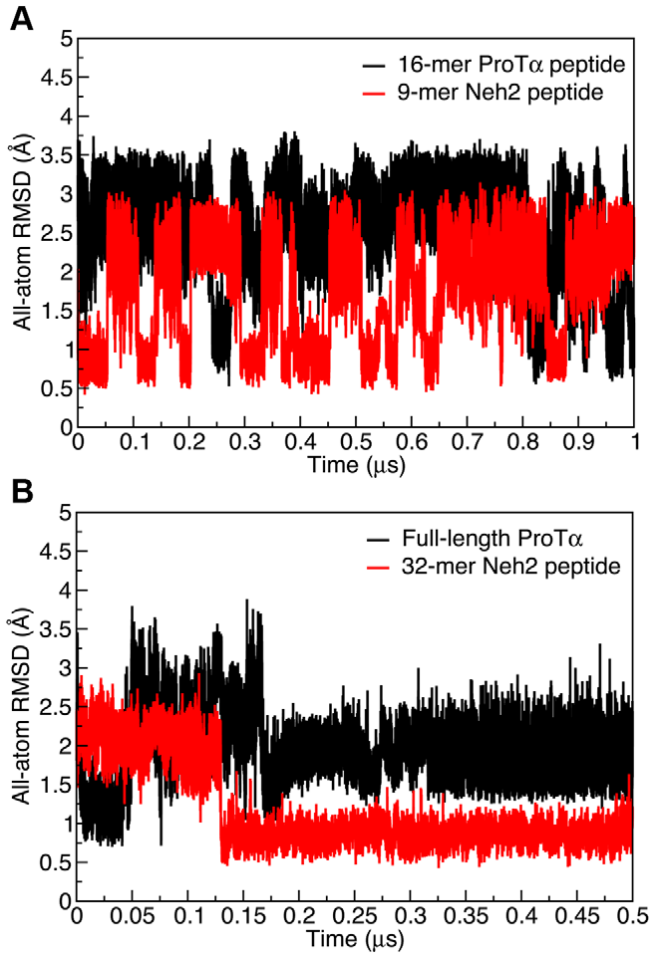
All-atom RMSD values between the MD and crystal structures. The RMSD values were computed by subtracting the all-atom distance matrix at time *t* of the MD trajectories from the reference distance matrix determined from the crystal structures of the ProTα and Neh2 peptides bound to Keap1 (PDB ids: 2Z32 and 1X2R respectively) [50], [55]. The distance matrices consisted of residues *i* through *i*+3 of the β-turn regions of the ProTα and Neh2 peptides determined from the crystal structures [50], [55].

We first determined the average distance-based RMSD values (Eq. 1) between the free-state MD structures of ProTα and Neh2 peptides and their corresponding Keap1 bound-state conformations (Table 2). To focus on the turn structure that is crucial for the Keap1 binding, only the four residues that are involved in the β-turn formation (NEEN and DEET of ProTα and Neh2, respectively) were included in the following calculations. The all-atom RMSD values plotted over the trajectories reveal that the β-turn segment in the ProTα peptide sampled conformations with ∼3 Å RMSD from the bound-state structure for the majority of the trajectory, and infrequently adopted lower RMSD (i.e. <1.0 Å) bound-state like conformations. In contrast, the 9-mer Neh2 peptide underwent conformational change between structures with ∼1.0 Å and ∼2.5 Å all-atom RMSD throughout the trajectory and adopted bound-state like conformations at multiple periods of time (Figure 2A; Video S1).

**Table 2:** Average distance-based RMSD values between the bound-state conformation and the MD structures.

System	C α (Å) ± sdev	Backbone^a^ (Å) ± sdev	All-atom (Å) ± sdev
16-mer ProTα peptide	1.17 ± 0.48	1.13 ± 0.40	2.47 ± 0.62
9-mer Neh2 peptide	1.02 ± 0.66	1.03 ± 0.59	1.73 ± 0.68
Full-length ProTα^b^	0.34 ± 0.12	0.44 ± 0.12	1.82 ± 0.25
32-mer Neh2 peptide^b^	0.18 ± 0.08	0.26 ± 0.07	0.85 ± 0.12

a Backbone atoms include N, C α and C.

b The last 0.1 µs of the trajectory was used in the RMSD calculations.

**Figure 3:**
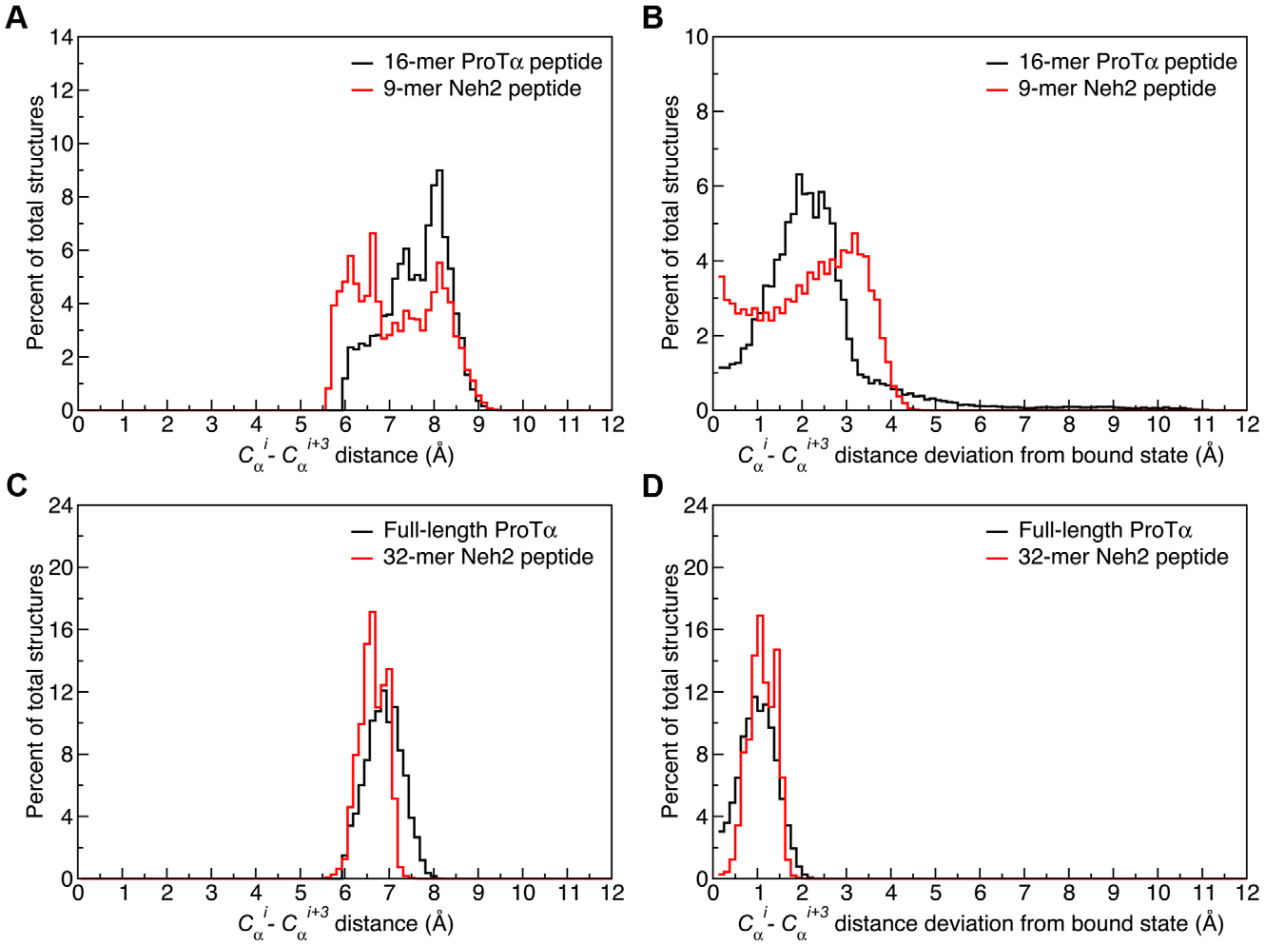
*C*α*^i^−C*α*^i+3^* distances and their deviations from their crystal structure distances. Panels A and C show the *C* α*^i^−C* α*^i+3^* distances. Panels B and D show the absolute deviations of *C* α*^i^−C* α*^i+3^* distances from the corresponding distances in the crystal structures. Data were collected over the full 1.0 µs trajectories for the crystal structure peptides and the last 0.1 µs for the full-length ProTα and 32-mer Neh2. Deviations were calculated for *C* α*^i^−C* α*^i+3^* pairs from the β-turns, determined from the crystal structures [50], [55], by subtraction of the *i* to *i*+3 distance at time *t* of the trajectory from the fixed distance of the corresponding atom pair from the crystal structures (PDB ids: 2Z32 and 1X2R) for ProTα and Neh2 respectively) [50], [55].

Next, we determined if defined β-turns were formed by the free-state peptides. A good indicator of β-turn formation is that the distance between the C_α_ atoms of residues *i* and *i*+3 (*C* α*^i^−C* α*^i+3^*) is less than 7 Å [72]. Based on this criterion, ∼28% of the structures from the 16-mer ProTα peptide trajectory adopted a β-turn conformation in that particular segment of the sequence, compared to ∼53% of the structures from the 9-mer Neh2 trajectory (Figure 3A). The same data set was also plotted in terms of deviation from their corresponding *C* α*^i^−C* α*^i+3^* values in the crystal structure (Figure 3B). The ProTα peptide had a single distribution of conformations, with an average *C* α*^i^−C* α*^i+3^* deviation of ∼2.2 Å from its bound state value (Figure 3B). In contrast, the *C* α*^i^−C* α*^i+3^* distance deviations for the Neh2 peptide showed that significant populations of structures had deviations of <1.0 Å and >3.0 Å (Figure 3B). This finding was consistent with the RMSD data, which showed that the 9-mer Neh2 peptide transitioned between ∼1 Å and ∼2.5 Å all-atom RMSD throughout the trajectory (Figure 2A). Importantly, the RMSD data and *C* α*^i^−C* α*^i+3^* distance distribution of the 9-mer Neh2 indicated that the free-state conformational ensemble of this peptide consists of both structures that closely resemble the bound-state β-turn conformation and ones that are comparably extended in that region (Video S1).

The above findings also indicate that during the 1-µs simulations, both the 16-mer ProTα and the 9-mer Neh2 peptides displayed intrinsic propensities of forming bound-state-like β-turn structures in the absence of Keap1. We realized that in the absence of Keap1, the peptides might not be long enough to form stable structures. To assess the contributions of residues outside the binding motifs in stabilizing the β-turn conformation, MD simulations of the full-length ProTα protein and a 32-mer Neh2 peptide were performed. Structural resemblance to their Keap1-bound states was gauged by the same parameters as above.

Figure 2B shows the distance-based all-atom RMSD values between the MD structures and the corresponding bound-state crystal structures of full-length ProTα and the 32-mer Neh2 peptide over 0.5-µs trajectories. Like above, the analyses focused on the four residues that are involved in the β-turn formation. Interestingly, both the full-length ProTα protein and the 32-mer Neh2 peptide achieved lower and more stable all-atom RMSDs than their shorter counterparts (Figure 2). Specifically, the full-length ProTα converged to an all-atom RMSD of ∼1 Å after a short period of simulation time despite having a starting structure with an RMSD ∼2.6 Å (Figure S1). The values of RMSD fluctuated between ∼0.75–3.75 Å in the first 0.18 µs and then stabilized at an all-atom RMSD around 2 Å for the remainder of the trajectory (Figure 2B). The 32-mer Neh2 peptide converged to an all-atom RMSD of less than 1 Å in about 0.13 µs and remained stable around that value for the rest of the trajectory (Figure 2B; Video S2). It is worth mentioning that the bound-state-like β-turn conformations formed by the full-length ProTα and the 32-mer Neh2 peptide closely resembled the ones adopted by their shorter peptide counterparts (Figure S2).

The *C* α*^i^−C* α*^i+3^* distances were also calculated to appraise the formation of β-turn structure during the simulations. The results show that during the last 0.1 µs of the full-length ProTα trajectory, ∼66% of the structures have the binding motif in β-turn conformations (*C* α*^i^−C* α*^i+3^* <7 Å), compared to ∼94% of the 32-mer Neh2 peptide structures (Figure 3C). It is noteworthy that both systems showed considerably smaller deviations from their bound-state *C* α*^i^−C* α*^i+3^* distances compared to their shorter counterparts (Figure 3D).

**Figure 4:**
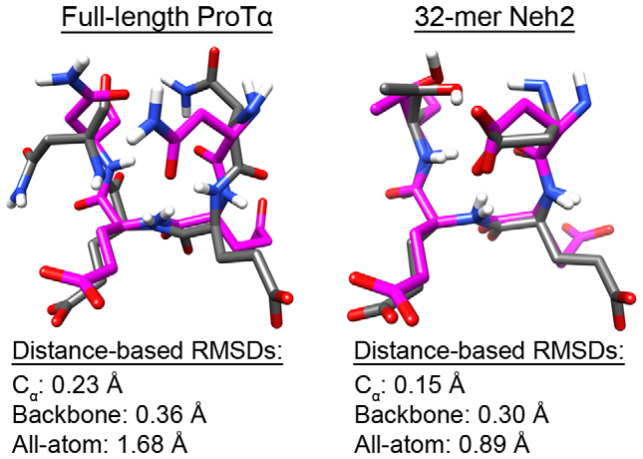
Overlay of the free and bound-state β-turns. Residues *i* through *i*+3 of the β-turns from the full-length ProT α and the 32-mer Neh2 MD structures were superimposed onto the corresponding residues from their bound state crystal structures. Cluster centroids from the last 0.1 µs of the MD simulations (grey) were superimposed onto the corresponding C_α_ atoms from the crystal structures (pink) of ProTα and Neh2 bound to Keap1 (PDB ids: 2Z32 and 1X2R respectively) [50], [55]. The single linkage clustering algorithm was used with a cutoff that included all structures from the last 0.1 µs. Hydrogens were added to the crystal structures for clarity. RMSD values were computed by subtracting the C_α_, backbone or all-atom distance matrix of the centroid structures from the reference distance matrix determined from the crystal structures of the ProTα and Neh2 peptides bound to Keap1 (PDB ids: 2Z32 and 1X2R respectively) [50], [55].

The superpositions of the cluster centroids of β-turn-forming residues from the MD simulations with their corresponding crystal structure atoms in Figure 4 further illustrate the structural similarities between the free and bound states for both ProTα and the Neh2 domain. The average distance-based RMSD values between the bound-state conformation and the MD structures were summarized in Table 2. Although both ProTα and Neh2 had average C α and backbone RMSDs below 0.5 Å, the RMSDs and standard deviations increase considerably when all atoms were considered. It is clear that the side chains were not all in their bound state-like conformations. Figure S3 shows the distributions of side chain torsion angles in the NEEN and DEET motifs of ProTα and Neh2, respectively. The results suggest that although the backbones of these two proteins have strong propensity of forming β-turn structure, the side chains within the turns are not restricted in torsion angle samplings. However, it is worthwhile to note that Thr-80 of Neh2 showed a clear preference for adopting a χ_1_ angle that closely resembled its bound state value (Figure S3). This is discussed further in the following section.

### Contributing factors to the β-turn propensities of ProTα and Neh2

**Figure 5:**
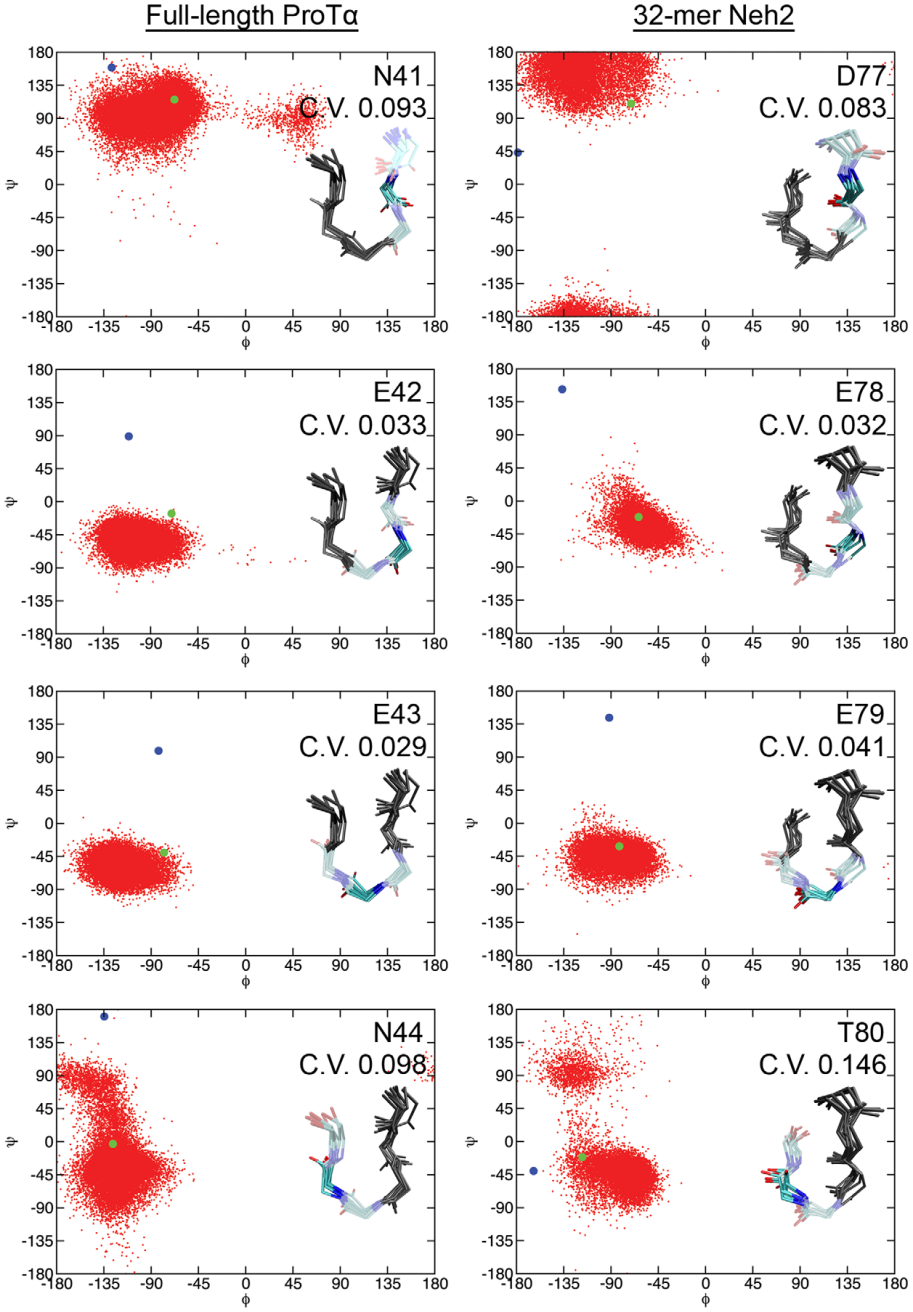
Ramachandran plots for residues *i* to *i*+3 of the β-turns from the MD and crystal structures. Red dots indicate the ϕ and ψ pair from the last 0.1 µs of the full-length ProTα and the 32-mer Neh2 trajectories. Blue circles indicate the angles of the starting structures. Green circles indicate the ϕ and ψ angle pair from the crystal structures (PDB ids: 2Z32 and 1X2R) [50], [55]. Circular variance (*C.V.*) values and overlaid licorice representation snapshots from the last 0.1 µs of the simulations illustrate backbone mobility within the β-turns of ProTα and Neh2. Average circular variance values were calculated over the last 0.1 µs of the full-length ProTα and the 32-mer Neh2 peptide MD trajectories using the method described by MacArthur & Thornton [73].

To determine residue-specific convergences of amino acids in the torsion angle space, backbone dihedral angles of the Keap1-binding β-turns from the MD trajectories were compared to their corresponding bound-state values. Since ProTα and Neh2 peptides bind to the same site on the Kelch domain of Keap1 and adopt structurally similar β-turns (Figure 1), their bound-state ϕ and ψ angles are comparable as expected (Figure 5). MD simulations show that, in their free states, both ProTα and Neh2 had preferences of sampling dihedral angles around their bound-state values (Figure 5). Circular variance (*C.V.*) measurements were used to quantify the spread of ϕ and ψ angles over the last 0.1 µs of the trajectories. Both ProTα and Neh2 had similar *C.V.* (Eq. 2) values for residues *i* to *i+2*, while ProTα displayed a slightly lower circular variance for residue *i+3* compared to that of Neh2 (Figure 5). Snapshots over the last 0.1 µs of the trajectories illustrate that the β-turns of Neh2 and ProTα had limited backbone flexibilities (Figure 5).

**Figure 6:**
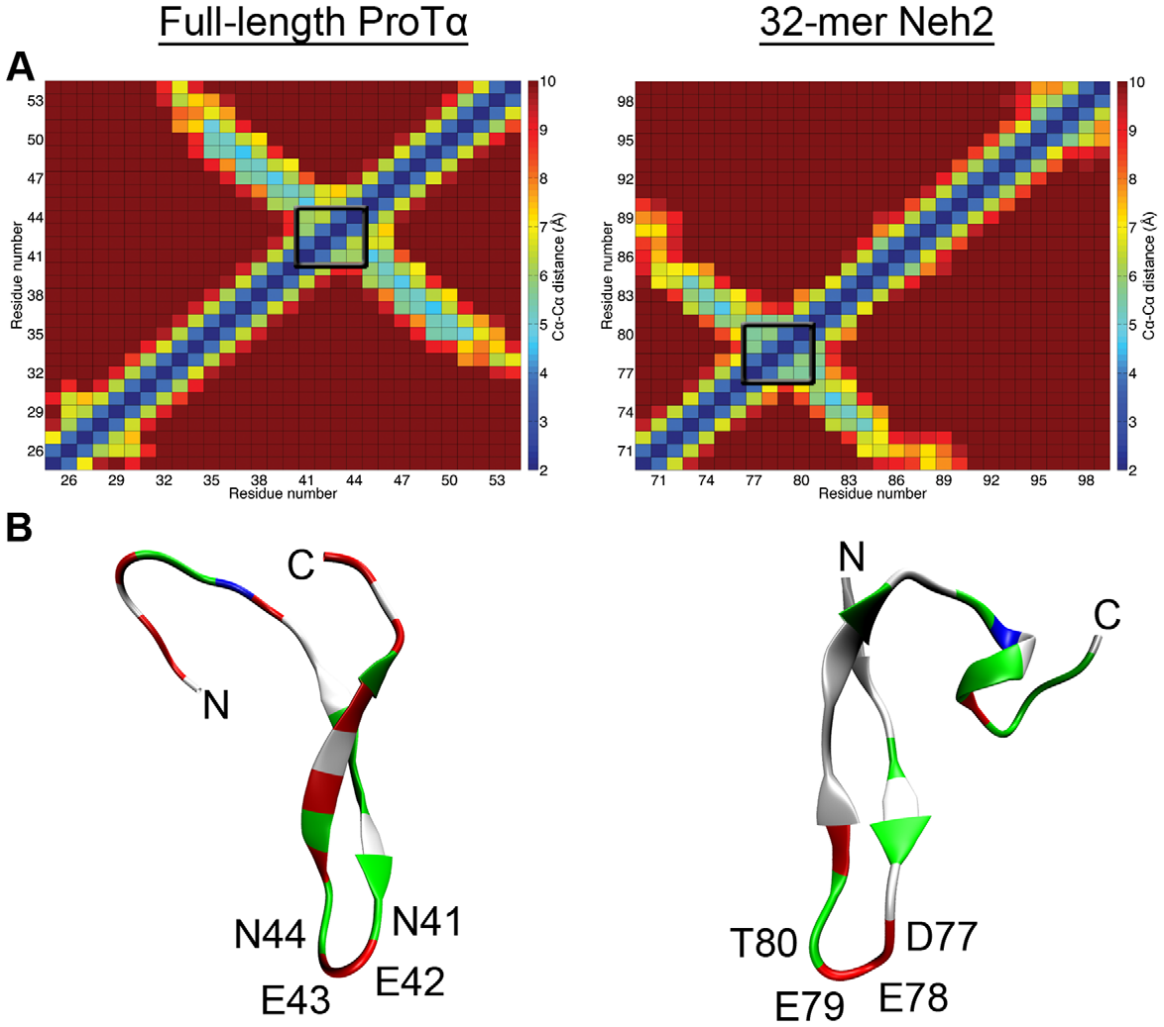
Cα*−*Cα contacts in the MD structures. A) Average C α*−*C α distances over the last 0.1 µs of the full-length ProTα and 32-mer Neh2 MD trajectories. Distances equal to or greater than 10 Å are colored dark red and distances equal to or less than 2 Å are colored dark blue. The *C* α*^i^−C* α*^i+3^* atoms of the β-turns are indicated by the black boxes. B) Cartoon B-Spline representations colored by residue type of ther Keap1 binding regions of full-length ProTα and 32-mer Neh2 cluster centroids from the last 0.1 µs of the MD simulations. The single linkage clustering algorithm was used with a cutoff that included all structures from the last 0.1 µs. Residues comprising the XEEXGE Keap1-binding motifs are labeled. Directionality is indicated with the N and C labels.

Contacts between C α*−*C α atom pairs during the last 0.1 µs of the simulations were also examined. The contact plots and structures from the MD simulations show that the β-turns formed by ProTα and Neh2 at their Keap1-binding sites stretched out in both directions to form antiparallel β-sheets (Figure 6). This finding was in good agreement with previous NMR results, which suggest that residual structures may exist in regions surrounding the Keap1-binding motifs of disordered ProTα and Neh2 [46], [78]. Interestingly, Neh2 has relatively higher ^1^H-^15^N heteronuclear NOE values in its Keap1-binding region, indicating a less dynamic free-state [46]. Furthermore, chemical shift index values indicative of β-strand structure and the observance of ^1^H, ^1^H NOEs between the adjacent strands also evidence that residues on either side of the ETGE motif of Neh2 form a short β-sheet [46]. Tong *et al*. suggested that interactions between the hydrophobic residues (Phe-74, Leu-76, Phe-83, and Pro-85) located on the β-strands may stabilize the antiparallel β-sheet structure [46]. This proposal is supported by the ITC data showing that a long Neh2 segment containing the ETGE motif bound to the Kelch domain of Keap1 with higher affinity than the 9-mer peptide used in the current study (K_d_≈8 nM vs K_d_≈182 nM) [46]. Similarly, Lo *et al.* [79] demonstrated that human Nrf2-derived 14-mer (LQLDEETGEFLPIQ) or 16-mer (AFFAQLQLDEETGEFL) peptides could compete with full-length Nrf2 for binding to Keap1 much better than a 10-mer peptide (LDEETGEFLP). Their ITC measurements showed that the human 16-mer Nrf2 peptide binds to the Kelch domain of Keap1 with K_d_≈20 nM, an affinity similar to that of the mouse homolog [79].

In this work, we have measured the binding affinities of mouse 16-mer ProTα and 9-mer Neh2 peptides to the Kelch domain using ITC (Table 3; Figure S4). The large and negative entropy changes of 16-mer ProTα and 9-mer Neh2 peptides upon binding to Keap1 (Table 3) clearly reflect the significant reduction in conformational entropy of the peptides due to the disorder-to-order transition upon binding. Even though the 16-mer ProTα and the 9-mer Neh2 peptides have similar binding affinity to the Kelch domain, the former interacts more weakly with Keap1 compared to the Neh2 peptide with the same length [79]. This observation is in good agreement with the lower propensity of the β-turn formation in ProTα that is critical for the binding revealed by our MD simulations. It is noteworthy that unlike Neh2, ProTα lacks comparable hydrophobic content in the region surrounding the Keap1-binding motifs (Table 1). The deficiency in hydrophobic interactions may also account for the lower binding affinity between ProTα and Keap1.

**Table 3:** Thermodynamic parameters for the binding of ProTα and Neh2 peptides to the Kelch domain of Keap1.

Peptide	n^a^	K_a_ b(10^6^ M^−1^)	ΔH^b^(kcal/mol)	TΔS^b^ (kcal/mol)	ΔG^b^(kcal/mol)
16-mer ProTα peptide	1.03	2.4 ± 0.1	−18.9 ± 0.1	−10.20	−8.70 ± 0.02
9-mer Neh2 peptide	1.02	3.7 ± 0.1	−19.0 ± 0.1	−10.05	−8.95 ± 0.02

a Binding stoichiometry.

b K_a_ is the binding constant. ΔH, ΔS and ΔG are the change in enthalpy, entropy and Gibbs free energy upon binding (at temperature T = 298 K), respectively.

Hydrogen bond analysis was conducted to help explain why the β-turns of ProTα and Neh2 converged to their bound state structures to different extents. Inspection of the MD structures from the last 0.1 µs of the simulations reveal that ProTα and Neh2 had different occurrence frequencies of hydrogen bonds within their Keap1-binding β-turns (Table 4). ProTα had at least one hydrogen bond present in only 14.3% of the structures, compared to a frequency of 98.6% for Neh2 (Table 4). The main differences arose from increased *i* to *i+3* and, to a lesser extent, *i* to *i+2* intra-turn hydrogen bonding in Neh2 compared to ProTα. For instance, hydrogen bonding between the side chains of Asp-77 and Thr-80 was observed in ∼80% of the Neh2 structures, while the corresponding side chain hydrogen bonding between Asn-41 and Asn-44 of ProTα was not observed in the MD trajectory (Table 4). The involvement of Thr-80 in intra-turn hydrogen bonds may explain why its χ_1_ angle closely resembled its bound state value (Figure S3). Furthermore, hydrogen bonding between the side chain of Asn-41 and the main chain of Asn-44 was observed in only 6.7% of the ProTα conformations, while, in the Neh2 trajectory, over 77.5% of the conformations were found to have hydrogen bonding between the side chain of Asp-77 and the main chain of Thr-80 (Table 4). In addition, the side chain of Asp-77 and the main chain of Glu-79 in Neh2 also form hydrogen bond more frequently compared to the corresponding residues in ProTα (55% vs 0.4%) (Table 4). The result of this analysis suggested that the greater number and more frequent intra-turn hydrogen bonds formed by Neh2, particularly between the *i* and *i+3* residues, may explain why it adopts more stable bound-state-like structure than ProTα. Interestingly, this finding qualitatively agrees with the difference in the residue-specific turn potentials for the β-turns of ProTα and Neh2. Using a table of overall turn potentials for each amino acid determined by Hutchinson & Thornton [80], the turn potentials for residues in the *i* to *i+3* positions were summed. The NEEN and DEET sequences of Neh2 and ProTα had turn potentials of 4.87 and 5.03 respectively. The lower value for ProTα compared to Neh2 arose partly due to asparagine being slightly disfavored in position *i* compared to aspartic acid, but mainly because threonine was considerably more favored in position *i*+3 than asparagine.

**Table 4:** Frequencies of intra-turn hydrogen bond formations.

Atom involved	Full-length ProTα^a^	32-mer Neh2^a^
mc^b^ i to mc i+2		0.196%
mc i to mc i+3		3.844%
mc i to sc i+3		27.808%
mc i+2 to sc i+3	0.284%	0.204%
sc^c^ i to mc i+2	0.396%	55.368%
sc i to mc i+3	6.696%	77.524%
sc i+1 to mc i+2		0.428%
sc i to sc i+3		80.212%
sc i+2 to sc i+3	7.316%	0.572%
Intra-turn total	14.348%	98.644%

a Each frame from the last 0.1 µs of the mouse full-length ProTα and 32-mer Neh2 trajectories were used for the hydrogen bond calculations (25,000 structures). A hydrogen bond between a hydrogen donor (D–H) and a hydrogen acceptor (A) was judged to be formed when the DA distance (r) was less than 3.2 Å and the angle between the DA vector and the D–H bond (AD-H angle) was less than 35°.For clarity, only hydrogen bonds occur in more than 0.1% of the structures are listed and intra-residue hydrogen bonds are excluded.

b mc – main chain atoms.

c sc – side chain atoms.

As shown in Table 4, a large fraction of the intra-turn hydrogen bonds formed by Neh2 involve Thr-80. Studies reveal that mutating Thr-80 of Neh2 to alanine disrupts the interaction between these two proteins, making Nrf2 resistant to Keap1 mediated degradation. In contrast, a T80S mutant, which has the side chain hydroxyl group retained, behaved similarly to the wild type [79]. Interestingly, the phosphorylation of Thr-80 has also been shown to severely decrease binding of Neh2 to Keap1 [79]. The authors suggested that the negative charge introduced by the phosphorylation may disrupt the β-turn formation, preventing Neh2 from adopting a complementary structure to the binding site of Keap1 [79].

The attenuation of Keap1 binding when Thr-80 is mutated to alanine is likely due to the disruption of the β-turn structure. This idea is reinforced by our findings, which showed that the side chain of this residue is involved in the majority of intra-turn hydrogen bonds in the free state (Table 4). Moreover, residue-specific turn potential calculation also indicates that when the DEET sequence of Nrf2 is mutated to DEEA, the turn potential falls below that of the ProTα sequence to 4.72. Therefore, Thr-80 may act as a function switch, allowing Nrf2 activity to be regulated efficiently by phosphorylation [79], [81].

### Comparison of the mouse and human simulations

Finally, MD simulations were performed on the human homologs of full-length ProTα and the 32-mer Neh2 peptide (Table 1). The sequence alignments (Figure S5) indicate that there is a large percentage of sequence identity between the human and mouse versions of ProTα and Neh2. The human isoform 2 of ProTα used in this study contains 110 residues, which is shorter than the corresponding mouse sequence by one amino acid. The deletion site is located near the Keap1 binding region, immediately before the NEEN sequence. Besides the deletion, the human and mouse ProTα sequences are differ in only 5 other positions (Figure S5). For the 32-mer Neh2, there are three substitutions in the human sequence; one of them is located three residues upstream of the DEET β-turn. The MD simulations of human ProTα and Neh2 therefore serve as pseudo duplicates of the mouse trajectories owing to the high sequence identities between the human and mouse versions of these two proteins. Moreover, the single-residue changes (deletion in ProTα and substitution in Neh2) close to the β-turn sequences also allowed us to gauge the effects of mutations on the simulations.

The structure of a 16-mer human Neh2 peptide bound to human Keap1 (PDB id: 2FLU) [79] was compared to the structure of mouse Neh2-Keap1. Average distance-based RMSD calculations show that the residues comprising the β-turns in human and mouse Neh2 peptides adopt almost identical structures, with a backbone RMSD less than 0.1 Å in the bound-states [55], [79]. For ProTα, the crystal structure of human ProTα-Keap1 was not currently available. Therefore, for consistency, in the following calculations, we continued to use the mouse structures (PDB ids: 2Z32 and 1X2R) [50], [55] as the bound-state references for the human MD data.

Due to the intrinsically disordered nature of ProTα and Neh2, the initial structures used for the simulations are not well-defined. To avoid the potential bias of conformational sampling, starting structures used in the MD simulations of human ProTα and Neh2 were different from that used for the mouse. Considering the residues comprising the β-turns, the all-atom RMSDs between starting structures for the human and mouse sequences were 2.41 Å and 2.48 Å for the full-length ProTα proteins and the 32-mer Neh2 peptides, respectively.

Like the mouse versions, the β-turns of the human full-length ProTα and the 32-mer Neh2 peptide also converged to bound-state-like structures by the end of the trajectories (Figure S6). In the last 0.1 µs of the trajectories, both ProTα and Neh2 had *C* α*^i^−C* α*^i+3^* distance deviations around 1 Å from their mouse bound-state distances, with Neh2 having slightly closer *C* α*^i^−C* α*^i+3^* contacts (Figure S7). Interestingly, the 32-mer human Neh2 peptide adopted structures with about the same all-atom RMSDs to the bound-state conformation after a similar amount of simulation time compared to the mouse version (Figure S6 and Figure 2B). Meanwhile, the human ProTα was able to adopt structures with a lower all-atom RMSD to its bound state compared to its mouse counterpart (Figure S6 and Figure 2B).

The hydrogen bond analysis showed that, like the mouse homolog, human ProTα formed *i* to *i+3* hydrogen bonds less frequently compared to Neh2 (Table S1). For instance, hydrogen bonding between the side chains of Asn-40 and Asn-43 was observed in 24.2% of the ProTα structures compared to 63.7% for the corresponding Asp-77 and Thr-80 pair in Neh2 (Table S1). Furthermore, hydrogen bonding between the side chain of Asn-40 and the main chain of Asn-43 was observed in 61.3% of the ProTα conformations compared to 74.5% for the corresponding Asp-77 and Thr-80 pair in Neh2 (Table S1). The results from the human systems reinforce the notion that *i* to *i+3* hydrogen bonding between Asp-77 and Thr-80 of Neh2 might be more preferable than the corresponding Asn pair in ProTα.

Unlike the high similarities between the simulations of the mouse and the human Neh2, the intra-turn hydrogen bonding patterns of the human and mouse versions of ProTα were less consistent (Tables 4 and S1). Higher occurrences of hydrogen bonding between the main chains of *i* and *i+2* residues, as well as between the side chain and main chain of *i* and *i+3* were found in human ProTα. We speculate that the discrepancies reflect lower simulation convergence due to the less restricted conformation sampling of free-state ProTα [78]. However, the influence of starting structures and sequence differences cannot be ruled out. Further experimental studies are required to validate these findings.

### Conclusion

In this work we have investigated how ProTα and Neh2 interact with a common binding partner, the Kelch domain of Keap1 using 0.5–1.0 µs MD simulations. Our main findings are that the XEEXGE Keap1 binding motifs of ProTα and Neh2 in their free states possess propensities to form bound-state-like structure to different extents. Neh2 was found to form a defined β-turn more frequently than ProTα and had lower RMSD to its bound state conformation. This may be attributed to a larger number of and more stable intra-turn hydrogen bonds. In particular, hydrogen bonding between Asp-77 and Thr-80 of Neh2 might be more preferable than the corresponding Asn pair in ProTα. However, we cannot rule out that other factors, such as the lack of comparable hydrophobic content surrounding the Keap1 binding region of ProTα. This may also contribute to the more dynamic nature of ProTα and its lower propensity for adopting bound-state-like conformations.

Addressing whether ProTα and Neh2 bind to Keap1 through PSEs, coupled folding and binding or a combination of both mechanisms was challenging. To conclude that binding occurs via PSEs, the free and bound state conformations would have to be highly similar or identical. The definition of being highly similar can be ambiguous, while restricting the definition to identical structures seems too stringent. In any protein-protein interaction there are likely to be a certain amount of structural changes upon binding. In this case, the backbone atoms of the β-turns overlay well with the crystal structure backbones, especially for Neh2. However, the side chain orientations of some residues show considerable differences. It is clear that both mechanisms are at work to different extents. Because our data shows that the Keap1 binding regions of ProTα and Neh2 tend to form β-turns that have an obvious resemblance to their bound state conformations, we propose that binding occurs synergistically via a combination of PSEs and coupled folding and binding with a heavy bias towards PSEs, especially for Neh2.

## Supporting Information

Video S1 Transition of the 9-mer mouse Neh2 peptide from an extended to a bound-state-like β-turn conformation.
(MPG)

Video S2 Convergence of the 32-mer mouse Neh2 peptide to a bound-state-like β-turn conformation.
(MPG)

Figure S1 Overlays of the starting structure (grey) and crystal structure (pink) β-turns. Residues *i* through *i*+3 of the β-turns from the starting structures, generated in CNS [54], were superimposed onto the corresponding residues from their bound state crystal structures. The RMSD values were computed by subtracting the all-atom distance matrix of the starting structures from the reference distance matrix determined from the crystal structures of the ProTα and Neh2 peptides bound to Keap1 (PDB ids: 2Z32 and 1X2R respectively) [50], [55]. The distance matrices consisted of residues *i* through *i*+3 of the β-turn regions of the ProTα and Neh2 peptides determined from the crystal structures [50], [55]. The starting structures for human ProTα and Neh2 were compared to the mouse structures (PDB ids: 2Z32 and 1X2R) [50], [55] as their bound-state references. Hydrogen atoms were added for clarity.
(TIF)

Figure S2 Overlays of the β-turn structures from the 16-mer ProTα and 9-mer Neh2 MD simulations (white) with those from the longer sequence simulations (pink). The RMSD values were computed by subtracting the all-atom distance matrices. The distance matrices consisted of residues *i* through *i*+3 of the β-turn regions of the ProTα and Neh2 peptides determined from the crystal structures [50], [55]. Centroid structures from the shorter peptide simulations with lowest RMSDs to the bound state (820–830 ns and 630–640 ns from the ProTα and Neh2 simulations, respectively) were superimposed onto the corresponding centroid structures from the last 100 ns of the longer sequence simulations.
(TIF)

Figure S3 χ1 and χ2 angles from the MD and bound-state structures. Plots of the sidechain χ1 and χ2 angles for residues i to i+3 of the β-turns are shown. Red dots indicate the angles from the last 0.1 µs of the full-length ProTα and 32-mer Neh2 trajectories. Black dots indicate the angles from the crystal structures (PDB ids: 2Z32 and 1X2R) for ProTα and Neh2 respectively) [50], [55].
(TIF)

Figure S4 Isothermal titration calorimetry (ITC) measurements. Panels A and B correspond to titrations of 16-mer ProTα and 9-mer Neh2 peptide to the mouse Kelch domain of Keap1, respectively. (Upper) The raw data of two ITC experiments each performed at 25°C. (Lower) The integrated heat changes, corrected for the heat of dilution, and the fitted curve assuming single-site binding.
(TIF)

Figure S5 Sequence alignments of the mouse and human full-length ProTα and 32-mer Neh2 constructs generated using ClustalW XXL. The Blosum scoring matrix was used and gap penalties were set at their default values. Opening and end gap penalties were set to 10. Extending and separation gap penalties were set to 0.05.
(TIF)

Figure S6 All-atom RMSD values between the MD and crystal structures. The RMSD values were computed by subtracting the all-atom distance matrix at time *t* of the MD trajectories from the reference distance matrix determined from the crystal structures of the ProTα and Neh2 peptides bound to Keap1 (PDB ids: 2Z32 and 1X2R respectively) [50], [55]. The distance matrices consisted of residues *i* through *i*+3 of the β-turn regions of the ProTα and Neh2 peptides determined from the crystal structures [50], [55].
(TIF)

Figure S7 *C* α*^i^−C* α*^i+3^* distances and their deviations from their crystal structure distances. Panels A and B show the *C* α*^i^−C* α*^i+3^* distances and the deviations from the corresponding distances in the crystal structures respectively. Data was collected over the last 0.1 µs of the full-length human ProTα and human 32-mer Neh2 trajectories. Deviations were calculated for *C* α*^i^−C* α*^i+3^* pairs from the β-turns, determined from the mouse crystal structures [50], [55], by subtraction of the *i* to *i*+3 distance at time *t* of the trajectory from the fixed distance of the corresponding atom pair from the crystal structures (PDB ids: 2Z32 and 1X2R) for ProTα and Neh2 respectively) [50], [55].
(TIF)

Table S1 Frequencies of intra-turn hydrogen bond formations in full-length human ProTα and human 32-mer Neh2 trajectories.
(DOCX)
