# Supplementary figures and images for: Microsecond Molecular Dynamics Simulations of Intrinsically Disordered Proteins Involved in the Oxidative Stress Response
Source: PLoS One. 2011 Nov 18;6(11):e27371. doi: 10.1371/journal.pone.0027371 (PMC3220680; doi:10.1371/journal.pone.0027371)

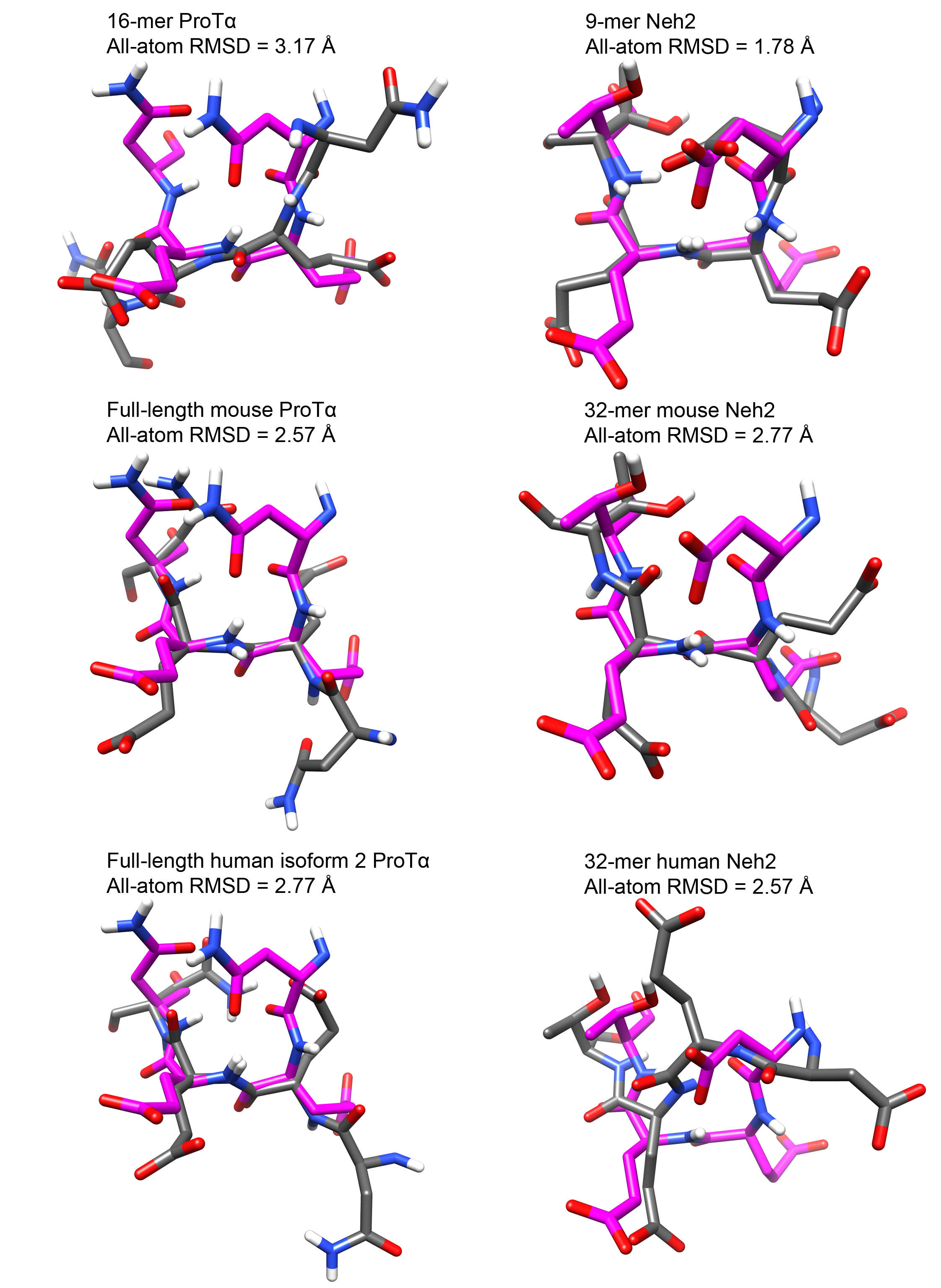

Supplement: Figure S1 — Overlays of the starting structure (grey) and crystal structure (pink) β-turns. Residues i through i+3 of the β-turns from the starting structures, generated in CNS [54], were superimposed onto the corresponding residues from their bound state crystal structures. The RMSD values were computed by subtracting the all-atom distance matrix of the starting structures from the reference distance matrix determined from the crystal structures of the ProTα and Neh2 peptides bound to Keap1 (PDB ids: 2Z32 and 1X2R respectively) [50], [55]. The distance matrices consisted of residues i through i+3 of the β-turn regions of the ProTα and Neh2 peptides determined from the crystal structures [50], [55]. The starting structures for human ProTα and Neh2 were compared to the mouse structures (PDB ids: 2Z32 and 1X2R) [50], [55] as their bound-state references. Hydrogen atoms were added for clarity. (TIF) [file pone.0027371.s003.tif]

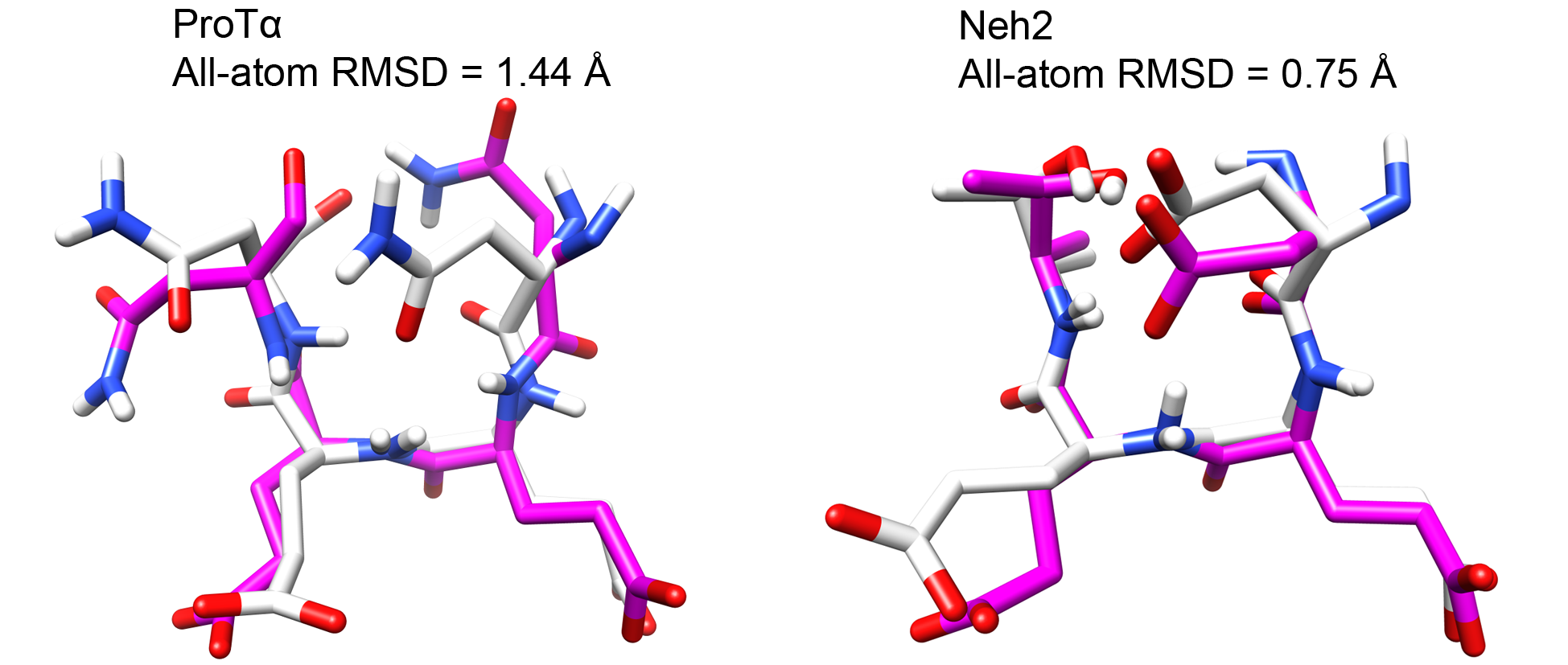

Supplement: Figure S2 — Overlays of the β-turn structures from the 16-mer ProTα and 9-mer Neh2 MD simulations (white) with those from the longer sequence simulations (pink). The RMSD values were computed by subtracting the all-atom distance matrices. The distance matrices consisted of residues i through i+3 of the β-turn regions of the ProTα and Neh2 peptides determined from the crystal structures [50], [55]. Centroid structures from the shorter peptide simulations with lowest RMSDs to the bound state (820–830 ns and 630–640 ns from the ProTα and Neh2 simulations, respectively) were superimposed onto the corresponding centroid structures from the last 100 ns of the longer sequence simulations. (TIF) [file pone.0027371.s004.tif]

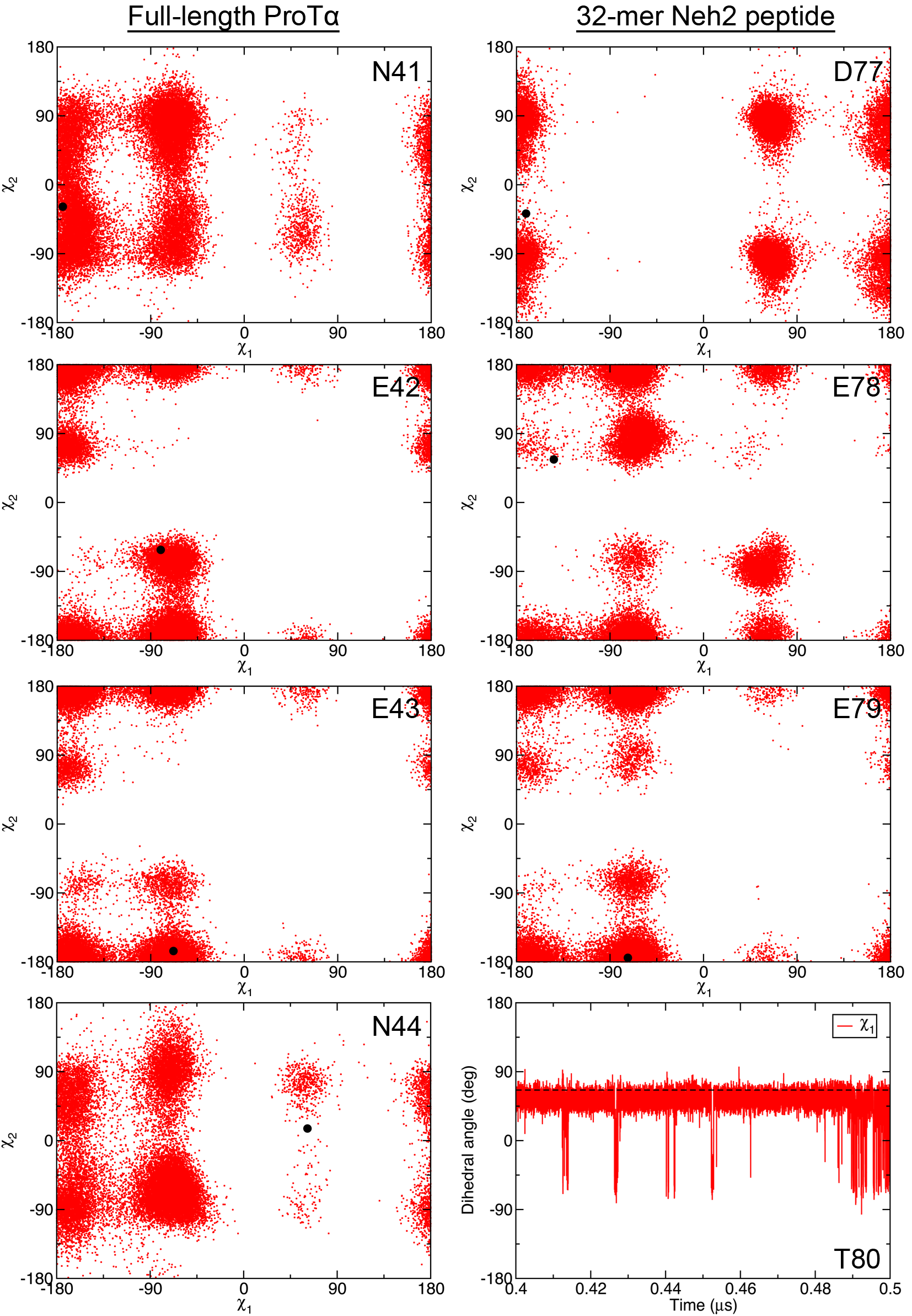

Supplement: Figure S3 — χ1 and χ2 angles from the MD and bound-state structures. Plots of the sidechain χ1 and χ2 angles for residues i to i+3 of the β-turns are shown. Red dots indicate the angles from the last 0.1 µs of the full-length ProTα and 32-mer Neh2 trajectories. Black dots indicate the angles from the crystal structures (PDB ids: 2Z32 and 1X2R) for ProTα and Neh2 respectively) [50], [55]. (TIF) [file pone.0027371.s005.tif]

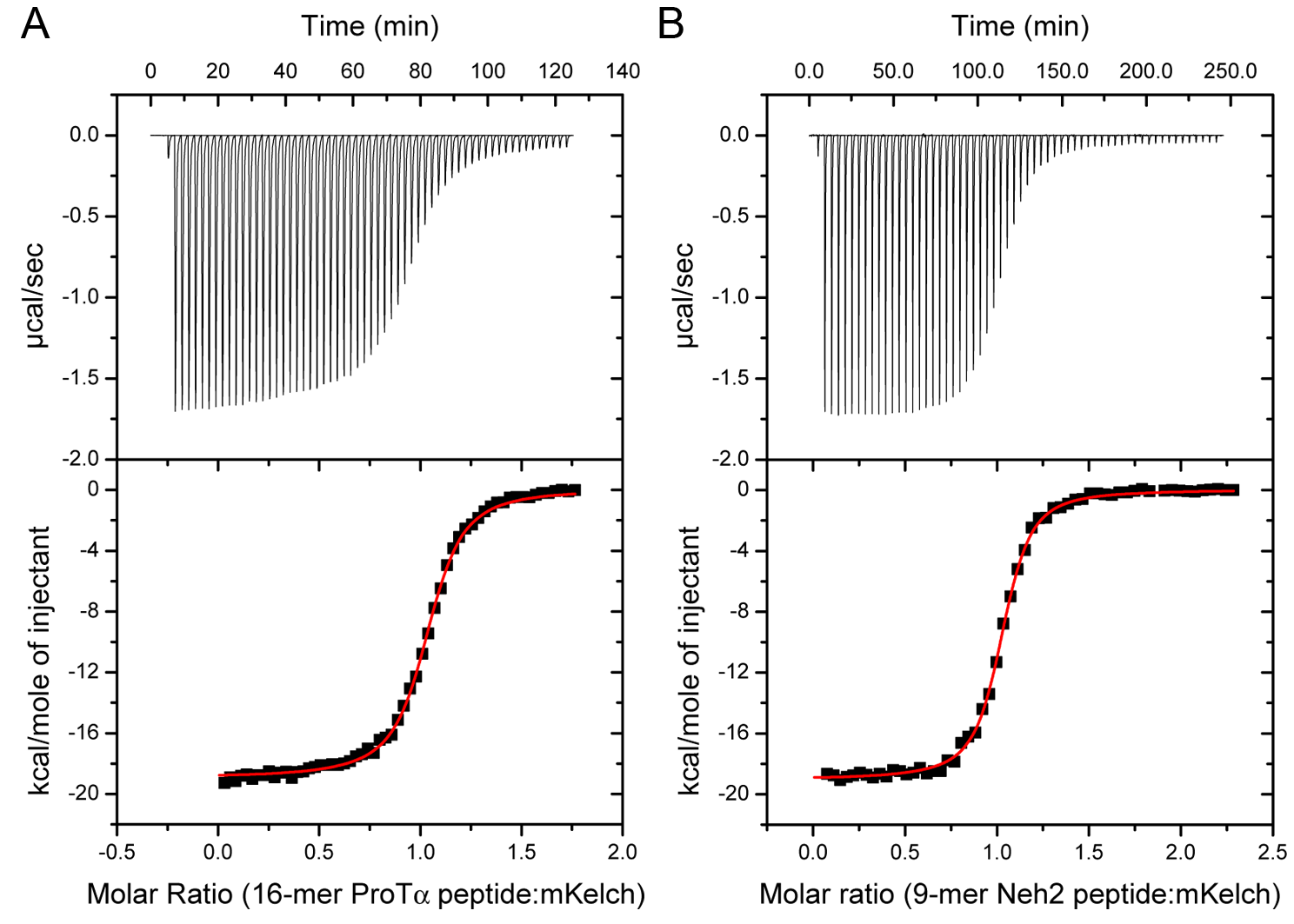

Supplement: Figure S4 — Isothermal titration calorimetry (ITC) measurements. Panels A and B correspond to titrations of 16-mer ProTα and 9-mer Neh2 peptide to the mouse Kelch domain of Keap1, respectively. (Upper) The raw data of two ITC experiments each performed at 25°C. (Lower) The integrated heat changes, corrected for the heat of dilution, and the fitted curve assuming single-site binding. (TIF) [file pone.0027371.s006.tif]

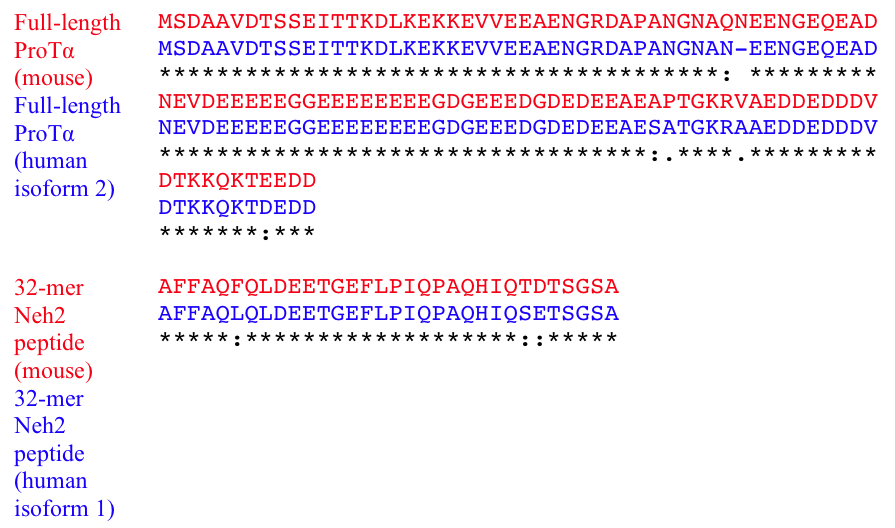

Supplement: Figure S5 — Sequence alignments of the mouse and human full-length ProTα and 32-mer Neh2 constructs generated using ClustalW XXL. The Blosum scoring matrix was used and gap penalties were set at their default values. Opening and end gap penalties were set to 10. Extending and separation gap penalties were set to 0.05. (TIF) [file pone.0027371.s007.tif]

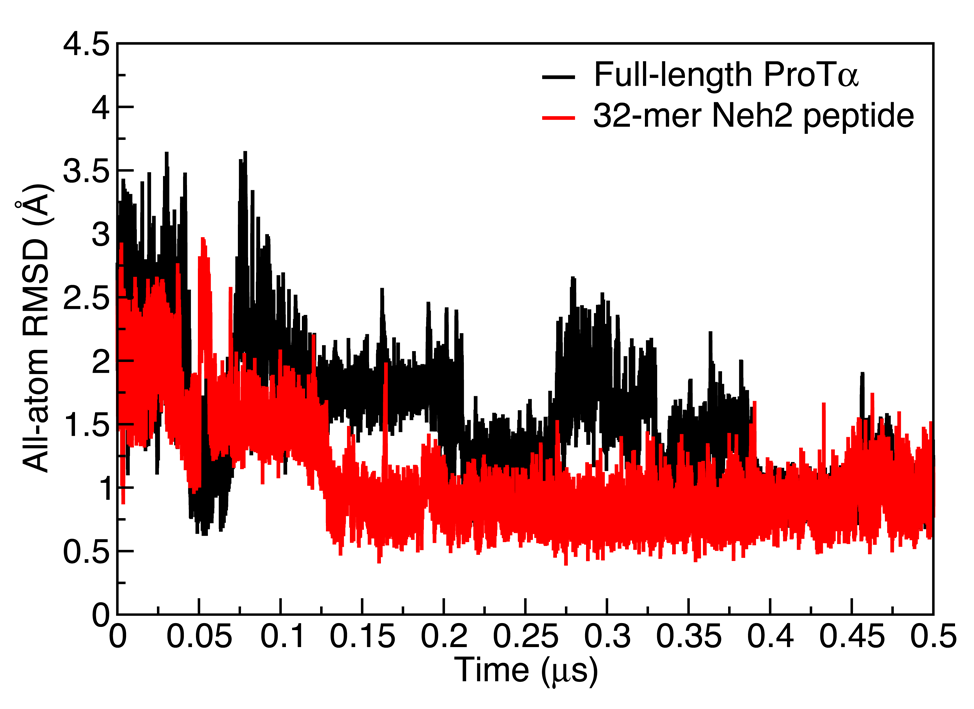

Supplement: Figure S6 — All-atom RMSD values between the MD and crystal structures. The RMSD values were computed by subtracting the all-atom distance matrix at time t of the MD trajectories from the reference distance matrix determined from the crystal structures of the ProTα and Neh2 peptides bound to Keap1 (PDB ids: 2Z32 and 1X2R respectively) [50], [55]. The distance matrices consisted of residues i through i+3 of the β-turn regions of the ProTα and Neh2 peptides determined from the crystal structures [50], [55]. (TIF) [file pone.0027371.s008.tif]

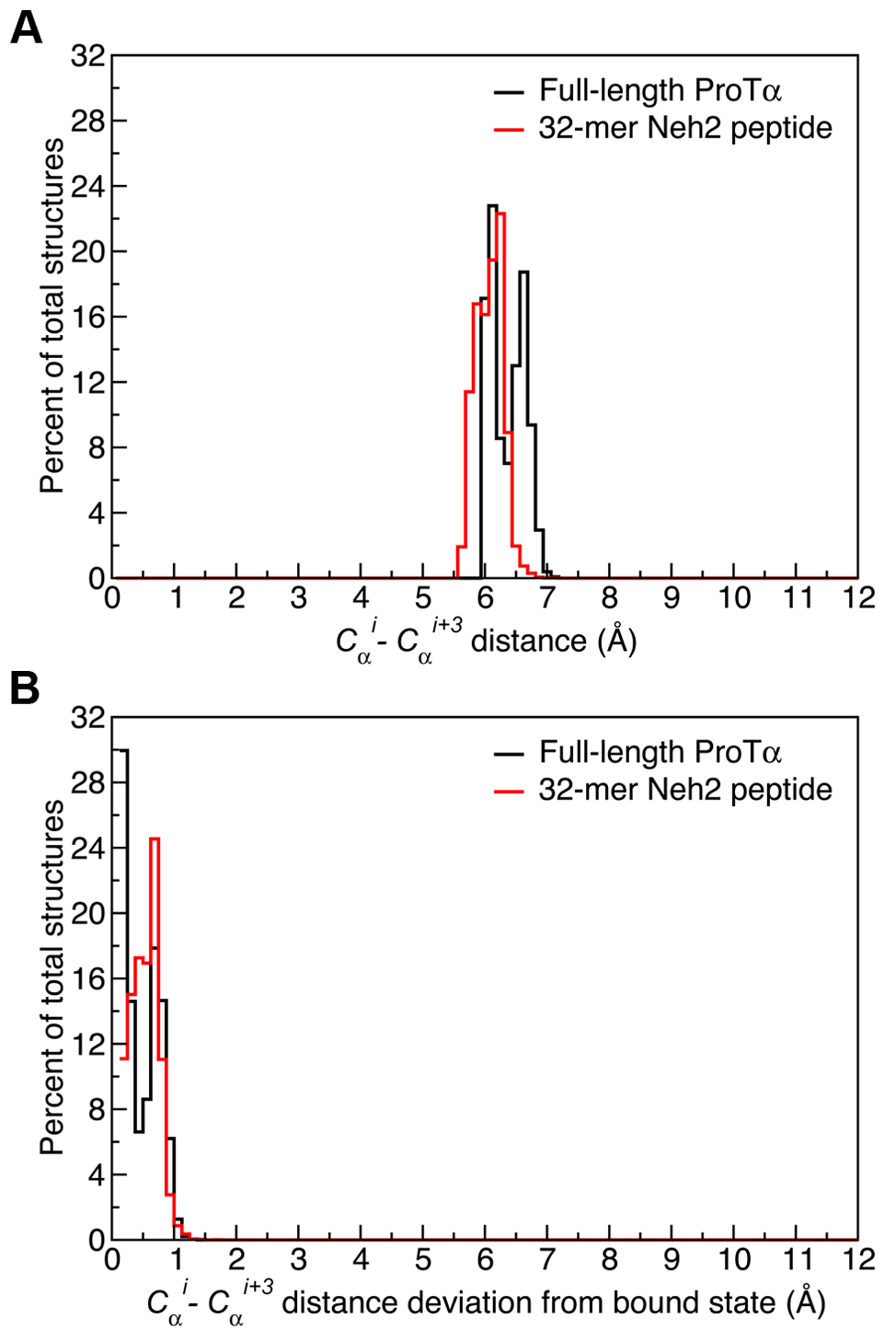

Supplement: Figure S7 — C αi−C αi+3 distances and their deviations from their crystal structure distances. Panels A and B show the C αi−C αi+3 distances and the deviations from the corresponding distances in the crystal structures respectively. Data was collected over the last 0.1 µs of the full-length human ProTα and human 32-mer Neh2 trajectories. Deviations were calculated for C αi−C αi+3 pairs from the β-turns, determined from the mouse crystal structures [50], [55], by subtraction of the i to i+3 distance at time t of the trajectory from the fixed distance of the corresponding atom pair from the crystal structures (PDB ids: 2Z32 and 1X2R) for ProTα and Neh2 respectively) [50], [55]. (TIF) [file pone.0027371.s009.tif]
