# Supplementary material for: Microsecond Molecular Dynamics Simulations of Intrinsically Disordered Proteins Involved in the Oxidative Stress Response
Source: PLoS One. 2011 Nov 18;6(11):e27371. doi: 10.1371/journal.pone.0027371 (PMC3220680; doi:10.1371/journal.pone.0027371)
Supplement: Table S1 — Frequencies of intra-turn hydrogen bond formations in full-length human ProTα and human 32-mer Neh2 trajectories. (DOCX) [file pone.0027371.s010.docx]

**Table S1. Frequencies of intra-turn hydrogen bond formations**

| Atoms involved | Full-length ProTα^a^ | 32-mer Neh2^a^ |
| --- | --- | --- |
| mc i to mc i+2 | 85.124% | 0.16% |
| mc i to mc i+3 |  | 1.744% |
| mc i to sc i+3 |  | 15.884% |
| mc i+1 to sc i |  | 1.708% |
| mc i+1 to sc i+2 |  | 0.56% |
| sc i to mc i+2 | 6.828% | 49.164% |
| sc i to mc i+3 | 61.272% | 74.504% |
| sc i+1 to mc i+2 |  | 0.328% |
| sc i+2 to mc i+3 | 0.112% |  |
| sc i to sc i+3 | 24.172% | 63.74% |
| sc i+2 to sc i+3 | 0.14% | 0.536% |
| Intra-turn total | 96.844% | 95.248% |

^a^ Each frame from the last 0.1 µs of the human full-length ProTα and 32-mer Neh2 trajectories were used for the hydrogen bond calculations (25 000 structures). A hydrogen bond between a hydrogen donor (D-H) and a hydrogen acceptor (A) was judged to be formed when the DA distance (r) was less than 3.2 Å and the angle between the DA vector and the D-H bond (AD-H angle) was less than 35°. For clarity, only hydrogen bonds occur in more than 0.1% of the structures are listed and intra-residue hydrogen bonds are excluded.

^b^ mc – main chain atoms

^c^ sc – side chain atoms
